# Genomic insights into body size evolution in Carnivora support Peto’s paradox

**DOI:** 10.1186/s12864-021-07732-w

**Published:** 2021-06-09

**Authors:** Xin Huang, Di Sun, Tianzhen Wu, Xing Liu, Shixia Xu, Guang Yang

**Affiliations:** Jiangsu Key Laboratory for Biodiversity and Biotechnology, College of Life Sciences, Nanjing Normal University, 210023 Nanjing, China

**Keywords:** Carnivora, Body size associated genes, Peto’s paradox, Rapidly evolving genes, Fixed amino acid changes

## Abstract

**Background:**

The range of body sizes in Carnivora is unparalleled in any other mammalian order—the heaviest species is 130,000 times heavier than the lightest and the longest species is 50 times longer than the shortest. However, the molecular mechanisms underlying these huge differences in body size have not been explored.

**Results:**

Herein, we performed a comparative genomics analysis of 20 carnivores to explore the evolutionary basis of the order’s great variations in body size. Phylogenetic generalized least squares (PGLS) revealed that 337 genes were significantly related to both head body length and body mass; these genes were defined as body size associated genes (BSAGs). Fourteen positively-related BSAGs were found to be associated with obesity, and three of these were under rapid evolution in the extremely large carnivores, suggesting that these obesity-related BSAGs might have driven the body size expansion in carnivores. Interestingly, 100 BSAGs were statistically significantly enriched in cancer control in carnivores, and 15 of which were found to be under rapid evolution in extremely large carnivores. These results suggested that large carnivores might have evolved an effective mechanism to resist cancer, which could be regarded as molecular evidence to support Peto’s paradox. For small carnivores, we identified 15 rapidly evolving genes and found six genes with fixed amino acid changes that were reported to reduce body size.

**Conclusions:**

This study brings new insights into the molecular mechanisms that drove the diversifying evolution of body size in carnivores, and provides new target genes for exploring the mysteries of body size evolution in mammals.

**Supplementary Information:**

The online version contains supplementary material available at 10.1186/s12864-021-07732-w.

## Background

Carnivores, or the mammalian order Carnivora, feed primarily or exclusively on animal matter. They represent a highly diverse and successful group of mammals, and are at the top of the food chain. The order contains a total of 280 species in 11 families [1], which are widely distributed all over the world, covering most of the major land masses, rivers, and all of the oceans. Carnivores are well-known for their dietetic preferences, carnassial dentition, skull shape and body size [2].

Body size is closely related to factors such as habitat, life history, metabolism and risk of extinction [3]. Previous studies revealed that the mass-specific basal metabolic rate of carnivores decreased with increasing body mass [4–6]. In terms of feeding habits, prey size and diversity increase with body size in predatory carnivores. Interestingly, a statistical analysis showed that, among terrestrial carnivores, the herbivorous species are relatively large, while the insectivorous species are relatively small [7]. A typical case, the Ursidae, may have increased in size due to their diet, which includes a high proportion of fruits and vegetation [8]. Importantly, the range of carnivore body size is unparalleled in any other mammalian orders [9, 10]. The largest carnivore—the male southern elephant seal (*Mirounga leonina*)—is more than 4,000 kg in body mass and over 5.8 m in length [11, 12], whereas the smallest carnivore—the least weasel (*Mustela nivalis*)—is only 29 g in body mass and 0.114 m in length [13, 14]. The difference between these two species is huge—over 130,000-fold in body mass and 50-fold in length—making the carnivores a good target for investigating the mechanism of mammalian body size evolution.

The formation of these highly discrepant body sizes of carnivores is probably a manifestation of adapting to their respective niche—i.e. each size has its own ecological advantages. Small carnivores have better reproductive efficiency, access to a wider variety of food and a greater ability to respond to environmental emergencies than do larger carnivores [15–17]. For example, small carnivores in Mustelidae and Viverridae adapted to exploit small rodent prey and invertebrates. Their smaller body allows them to move swiftly enough to follow and pounce on prey and be inconspicuous when hunting in open vegetation [10].

In contrast, a large body size can also bring a multitude of benefits, including the ability to exploit vast food resources, increased competitiveness, increased defense against predation, and extended longevity [18, 19]. However, a larger body size means more cells, which in turn theoretically means a higher risk of cancer, assuming that each cell has an equal risk of mutating [20]. Notably, some extremely large carnivores such as the walrus (*Odobenus rosmarus*) and polar bear (*Ursus maritimus*), which can live for more than 40 years, were not found to have a higher risk of cancer than smaller species in Mustelidae, which have a lifespan of only several years [21]. This phenomenon is well-known as Peto’s paradox [20, 22]. Their highly variable body sizes make carnivores an excellent group for testing Peto’s paradox at the molecular level.

Until now, the molecular mechanisms regulating the body size of carnivores remain poorly explored. Previous studies mainly focused on the intraspecific variation in body size, especially in the domestic dog (*Canis lupus familiaris*). A recent study showed that variants in *IGF1*, *COL11A2*, *ITGA10* and *ADAMTS17* contributed to height and segregate within specific dog breeds [23]. The constantly updated high-quality genomes of carnivores provide new opportunities for studying the mechanism behind the huge variations in body size among carnivores. In the present study, a comparative genomic analysis was performed on 20 high-quality carnivore genomes. First, phylogenetic generalized least squares (PGLS) methods were used to scan for body-size-associated genes (BSAGs). Then, we determined the rapidly evolving genes (REGs) in small or extremely large carnivores and identified fixed amino acid changes in different body size groups. Finally, we tested whether selective pressure variation on cancer-related BSAGs among different carnivores partly verify Peto’s paradox. Since this study focused on a group of wild animals including many threatened or endangered species, and it is not possible or practical to collect fresh tissue samples. Therefore, no differentially expressed gene-related analysis was performed in this study. Using the results of from the above analyses, we hope to provide some novel insights into the molecular mechanism behind body size evolution in carnivores and mammals.

## Results

### Genome-scanning of BSAGs and functional enrichment

**Fig. 1:**
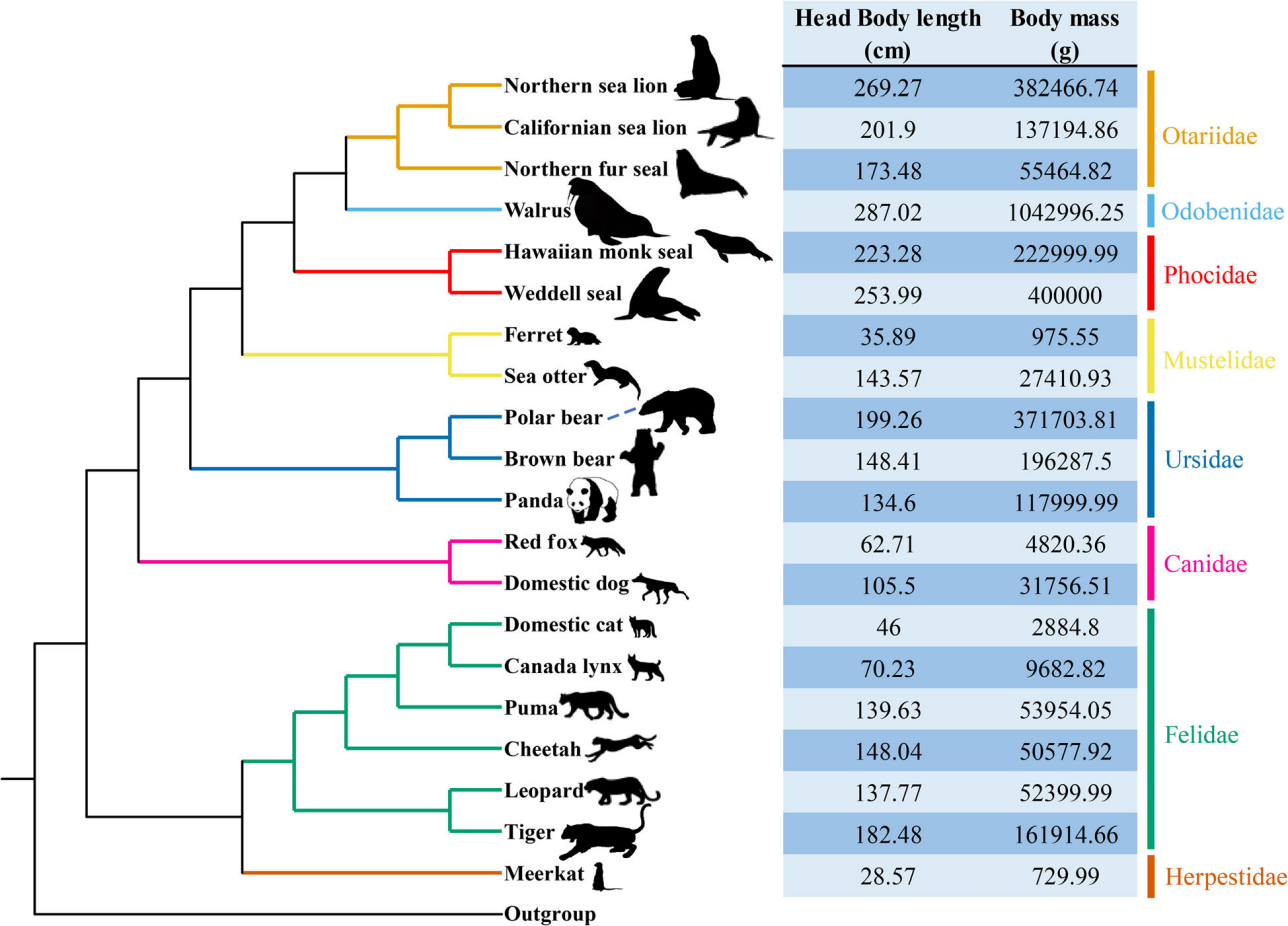
Phylogenetic tree and phenotype data on the head body length (cm) and body mass (g) of 20 carnivores. All phenotype data were collected from the PanTHERIA database and all silhouettes are reproduced from PHYLOPIC (http://phylopic.org/)

A total of 6,667 one-to-one orthologous genes were identified in the genomes of the 20 carnivores and one cow (Fig. 1; Table S1) using Orthofinder and our in-house Perl scripts. PGLS revealed that 1,132 and 668 genes were significantly associated with head body length and body mass, respectively. The two-step calibration procedure (*P value.all/robust/max* < 0.05) revealed that 337 of these genes were significantly related to both head body length and body mass; these were defined as body-size-associated genes (BSAGs; Table S2). According to the tendency or the correlation slope, 256 genes showed a positive correlation and 81 genes exhibited a negative correlation.

**Fig. 2:**
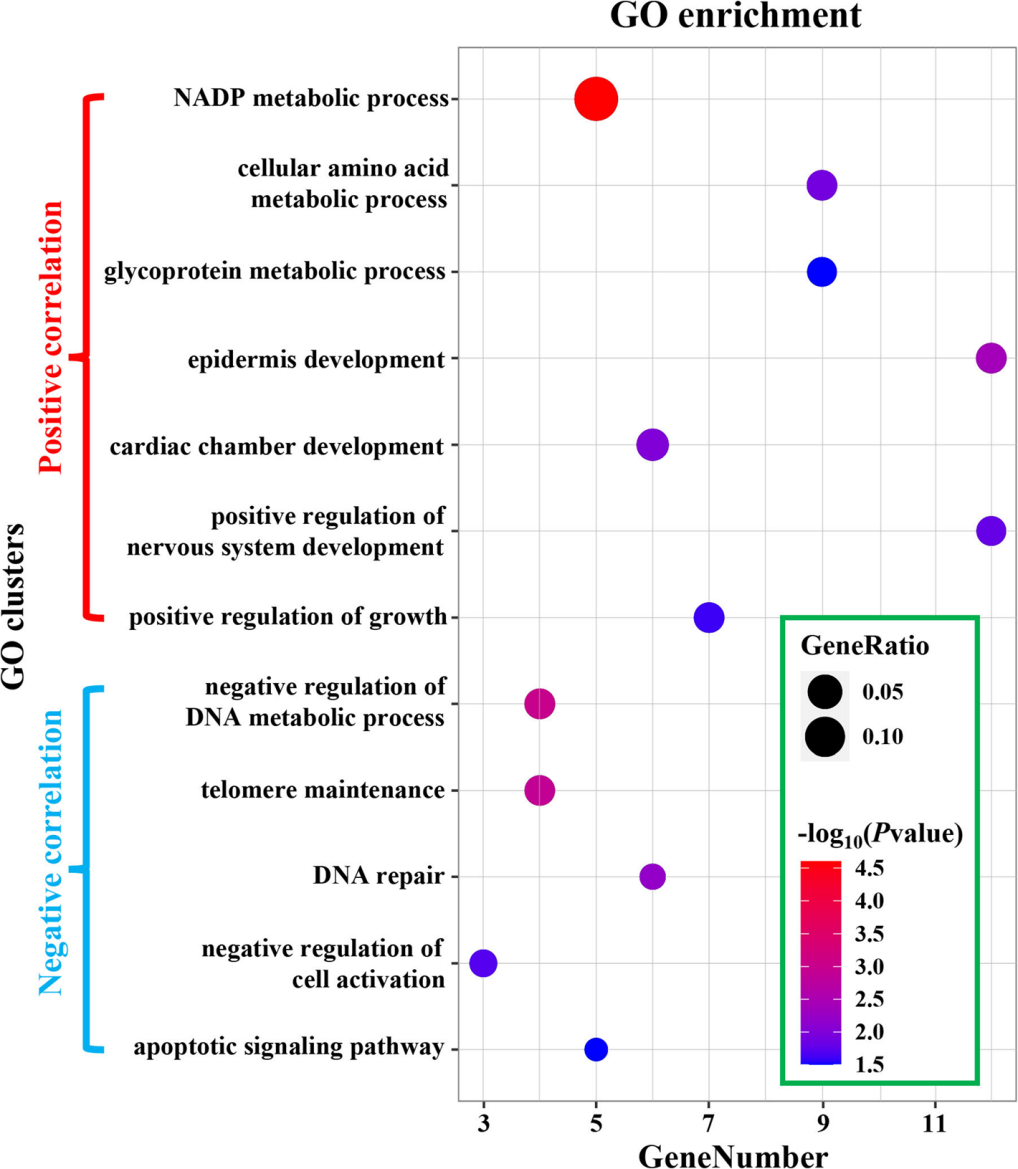
Functional analysis of BSAGs based on GO enrichments. GO clusters with a *P value* < 0.05 were significantly enriched in positively- or negatively-correlated BSAGs. The circle size represents the “GeneRatio,” or the ratio of enriched genes to all genes in the cluster. The color of the circles indicates the *P value* of GO clusters. The left y-axis represents the GO clusters, and the x-axis shows the gene numbers

Of the 256 positively-correlated BSAGs, functional enrichment analyses revealed that 62.9 % (161/256) were significantly enriched (*P value* < 0.05) in 164 GO terms (Table S3). 28.6 % (46/161) were annotated with metabolic processes, such as “NADP metabolic process,” “glycoprotein metabolic process” and “cellular amino acid metabolic process” (Fig. 2). 32.3 % (52/161) were significantly enriched in GO categories associated with growth and development, including “positive regulation of growth,” “cardiac chamber development,” “positive regulation of nervous system development” and “epidermis development” (Fig. 2). For instance, seven genes (*ADAM10*, *DBN1*, *NTRK3*, *PPIB*, *MAP2K5*, *WNT2* and *ZFPM2*) that play key roles in maintaining normal organ or body development were enriched in the GO term “positive regulation of growth.” However, due to the scattered functions of these BSAGs, there were only 21 (8.2 %) genes significantly enriched in KEGG pathways (Table S3)—e.g. “cytokine-cytokine receptor interaction” and “fanconi anemia pathway.”

Of the negatively-correlated BSAGs, 29.6 % (24/81) genes were classified into GO categories such as “telomere maintenance,” “DNA repair,” “negative regulation of DNA metabolic process,” “negative regulation of cell activation” and “apoptotic signaling pathway” (Fig. 2; Table S3). Mapping these negatively-correlated genes onto the KEGG database did not yield any significantly-enriched terms.

**Fig. 3:**
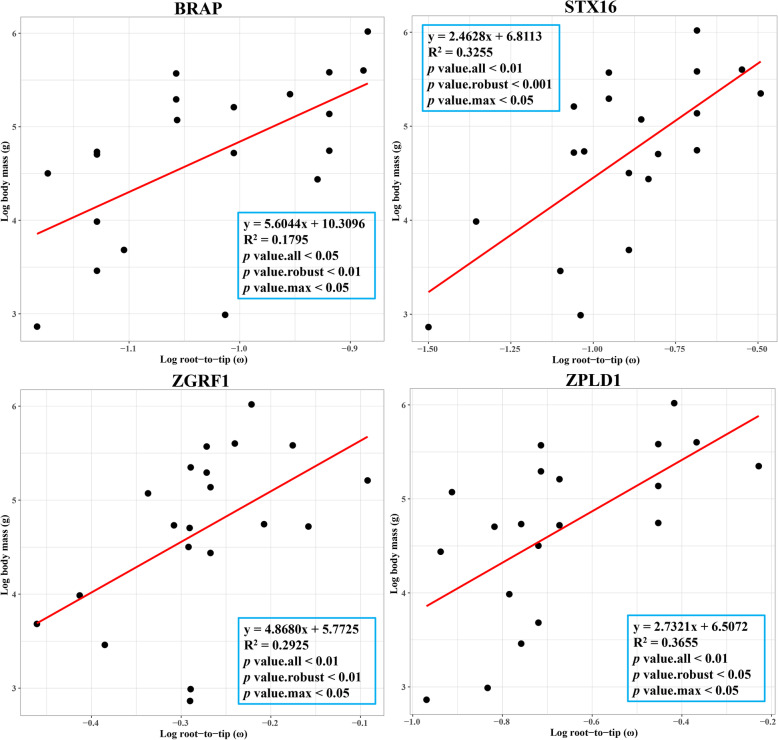
Regression analyses between root-to-tip (ω) and body mass (g) by PGLS in R for four obesity-related BSAGs: *BRAP*, *STX16*, *ZGRF1* and *ZPLD1*

We then manually looked up the biological functions of these BSAGs by searching the literature and multiple databases and identified 14 positively-correlated BSAGs (*BRAP*, *CHCHD5*, *CPT1C*, *GPR1*, *LDLR*, *MAP2K5*, *PLEKHS1*, *SLC30A8*, *ST3GAL2*, *STX16*, *ZFHX3*, *ZGRF1*, *ZNF395* and *ZPLD1*) that were associated with “obesity” (Table S4), which is a manifestation of an enlarged-body-sized phenotype. For instance, a significant positive association between log _(root−to−tip ω)_ and log _(body mass)_ was tested in *BRAP, STX16, ZGRF1* and *ZPLD1* (Fig. 3), and all four genes were reported to cause obesity in humans. Furthermore, we also found 100 BSAGs that were associated with cancer control, including “DNA repair,” “cell cycle control, apoptosis, adhesion and autophagy” and “immune response” (Table S5). Among these, a total of 21 BSAGs (*ADAM11*, *APC*, *BRCA2*, *CDH11*, *CERS2*, *DSC3*, *DTWD1*, *EPHB6*, *ERCC3*, *ERCC4*, *FANCC*, *HELQ*, *HRG*, *ING1*, *INTS6*, *POU6F2*, *STAG1*, *TEP1*, *TET1*, *TRMT2A* and *ZFHX3*) were identified to be tumor suppressors according to the literature or CGC database (Table 1). In addition, 18 genes were related to immunocyte immune response, development and maturation—e.g. *ITK* and *TNFRSF17* (Table S5).

**Table 1:** Twenty-one tumor suppressor genes that are significantly associated with the evolution of body size in Carnivora and their roles in cancer

Gene Symbol	Gene Name	Roles in Cancer
ADAM11^b^	ADAM metallopeptidase domain 11	Represents a candidate tumor suppressor gene for human breast cancer^c^
APC^a^	APC regulator of WNT signaling pathway	Encodes a tumor suppressor protein that acts as an antagonist of the Wnt signaling pathway and is involved in multiple processes, including cell adhesion and apoptosis^c^
BRCA2^a^	BRCA2 DNA repair associated	BRCA2 are involved in maintaining genome stability, specifically the homologous recombination pathway for double-stranded DNA repair^c^
CDH11^a^	cadherin 11	Encodes a type II classical cadherin from the cadherin superfamily, integral membrane proteins that mediate calcium-dependent cell-cell adhesion^c^
CERS2	ceramide synthase 2	Plays a role in the regulation of cell growth^c^ and suppresses tumor cell invasion [24]
DSC3	desmocollin 3	Has tumor suppressive activity through inhibition of AKT pathway in colorectal cancer [25]
DTWD1	DTW domain containing 1	A recently identified p53 target gene that inhibits cancer cell growth by reducing cyclin B1 expression [26]
EPHB6	EPH receptor B6	Encodes protein that influence cell adhesion and migration, and may suppress tumor invasion and metastasis^c^
ERCC3^a^	ERCC excision repair 3, TFIIH core complex helicase subunit	Encodes an ATP-dependent DNA helicase that functions in nucleotide excision repair^c^
ERCC4^a^	ERCC excision repair 4, endonuclease catalytic subunit	Encodes the protein that forms a complex with ERCC1 and is involved in the 5’ incision made during nucleotide excision repair^c^
FANCC^a^	FA complementation group C	Encodes the protein for complementation group C in Fanconi anemia, a genetic heterogeneous recessive disorder characterized by cytogenetic stability, increased chromosome breakage and defective DNA repair^c^
HELQ	helicase, POLQ like	Plays a critical role in replication-coupled DNA repair, germ cell maintenance and tumor suppression in mammals [27]
HRG^a^	histidine rich glycoprotein	Involves both inflammatory promoting effect in chronic disease and tumor suppression during the development of hepatocellular carcinoma [28]
ING1	inhibitor of growth family member 1	Encodes a tumor suppressor protein that can induce cell growth arrest and apoptosis and is a component of the p53 signaling pathway^c^
INTS6	integrator complex subunit 6	Encodes a DEAD box protein that is part of a complex that interacts with the C-terminus of RNA polymerase II and is involved in 3’ end processing of snRNAs. This gene is also a candidate tumor suppressor and is located in the critical loss of heterozygosity (LOH) region^c^
POU6F2	POU class 6 homeobox 2	Encodes a member of the POU protein family and is involved in Wilms tumor (WT) predisposition as a tumor suppressor^c^
STAG1^a^	stromal antigen 1	Plays a crucial role in the control of chromosome segregation during cell division as well as in DNA repair and replication [29]
TEP1^a, b^	telomerase associated protein 1	This gene product is a component of the ribonucleoprotein complex responsible for telomerase activity^c^
TET1^a^	tet methylcytosine dioxygenase 1	Encodes a demethylase that plays a role in the DNA methylation process and gene activation^c^
TRMT2A	tRNA methyltransferase 2 homolog A	Plays the inhibitory role in cell proliferation and cell cycle control [30]
ZFHX3^a^	zinc finger homeobox 3	Encodes the protein that transactive the cell cycle inhibitor cyclin-dependent kinase inhibitor 1 A; this gene reportedly functions as a tumor suppressor in several cancers^c^

^a^Source: Tumor suppressor genes that annotated by CGC database

^b^Source: Tumor suppressor genes that exhibits rapid evolution in extremely large carnivores

^c^Source: RefSeq

### REGs in different body sizes groups

“Branch model” implemented in Codeml of the program PAML 4.9e was used to identify rapidly evolving genes (REGs) in the small- and extremely large-body-sized groups (hereafter, any reference to a “small” or “large” group refers to body size). These results showed that divergent selective pressure might have acted on carnivores with contrasting body sizes.

In the small group, a total of 15 BSAGs were found to be under rapid evolution (Table S6). Among them, *CTLA4* (cytotoxic T-lymphocyte associated protein 4) is related to lower body mass index in humans, which fits into the small phenotype category. In the end, after false discovery rate (FDR) correction, only five REGs were still significant: *MAS1*, *CATSPERG*, *YTHDC2*, *SLC25A28* and *ADGRF2* (Table S6; Table S7).

By contrast, 60 REGs were placed in the extremely large group (Table S6). Fifteen genes were significantly enriched in growth and development (such as nerve, muscle and cardiac development) or phenotypic changes in body size (*ATP8B1*, *DIS3*, *POMGNT1*, *SLITRK5*, *ST3GAL2*, *TENM3*, *ZGRF1* and *ZPLD1*). Additionally, fifteen genes were associated with cancer control (Table S5); among these, three were related to immunity (*MAGT1*, *RFXANK* and *SKAP2*), two (*ADGRL3* and *TENM3*) were related to cell adhesion, and two (*ADAM11* and *TEP1*) were found to be tumor suppressor genes. Finally, 21 REGs maintained significance after correction for multiple testing using FDR (Table S6; Table S7).

### Fixed amino acid changes in the extremely small group

**Fig. 4:**
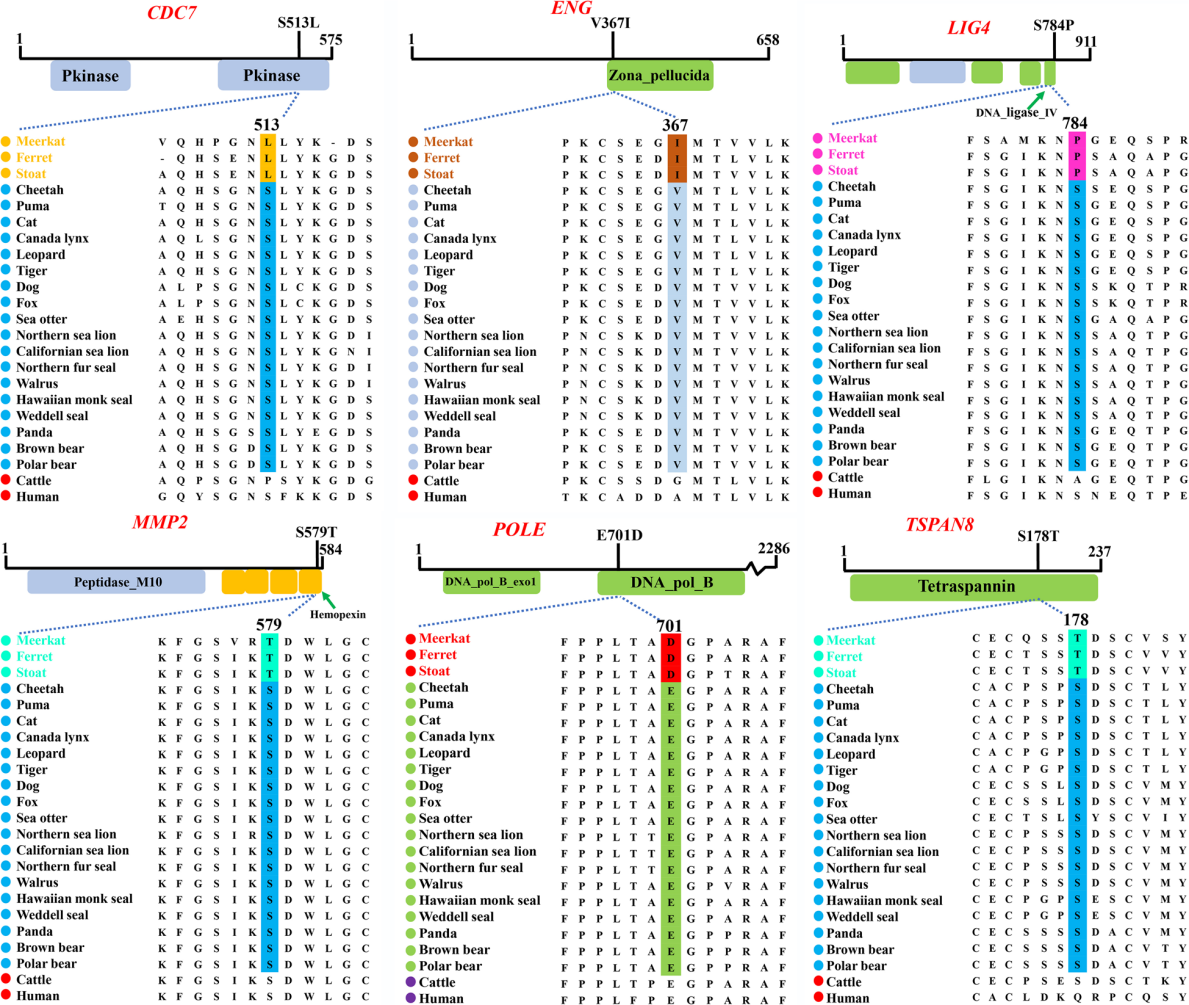
The fixed amino acid changes in extremely small carnivores observed in six genes related to the phenotype of reduced body size. Each of the six sites are located in a functional domain of the genes *CDC7*, *ENG, LIG4*, *MMP2*, *POLE* and *TSPAN8*

Identifying fixed amino acid changes in a certain group may help explain the molecular mechanism behind the occurrence of a specific phenotype. In the present study, we identified six fixed amino acid changes in six genes in extremely small carnivores (*CDC7*, *ENG*, *LIG4*, *MMP2*, *POLE* and *TSPAN8*; Fig. 4), but none in the small or extremely large carnivores. These sites were located in the functional domains of their respective proteins identified by Pfam. For instance, a unique change, S513L, was located in the protein kinase domain of *CDC7*, and another mutation (S784P) was found in the DNA ligase IV domain of *LIG4*; both of these genes were associated with the reduced-body-sized phenotype in humans or mice.

## Discussion

### Obesity-related genes contributing to increasing body size in carnivores

In Carnivora, some species in Pinnipedia and Fissipedia have evolved a relatively large body size, with some extremely large species weighing more than 350 kg, such as the walrus, northern sea lion, Weddell seal and polar bear. Due to their semi-aquatic life habits, species in Pinnipedia had evolved a large body size, which leads to an increased body surface area and reduction in rapid heat loss in water [31]. Similarly, the polar bear is the largest extant bear to adapt to the cold Arctic regions, weighing 372 kg on average [32]. Interestingly, polar bears and seals have relatively thick subcutaneous fat, which accounts for more than 30 % of their body weights, much more than that of other wild carnivores [33–35]. It has been suggested that the thick layer of fat covering pinnipeds and polar bears is an adaptation to the cold. In general, obesity refers to certain degree of overweight and a thick fat layer, which is caused by excessive fat accumulation [36]; a percent body fat ≥ 25 % for men and ≥ 30 % for women is an indicator of obesity [37].

In this study, we used PGLS to detect subtle variations among carnivores, as well as correlations between genes and phenotypes that are consistent across their phylogeny [38]. The root-to-tip *d*_N_/*d*_S_ method has been proved to be a powerful tool to detect gene-phenotype associations because it is more inclusive of evolutionary history of a locus and is therefore more suitable for regressions against phenotypic data from extant carnivores [38–40]. Thus, we could partly resolve the evolutionary mechanism of body size variation and identify key candidate genes that influence body size changes in Carnivora. Consequently, we found that variations in 14 positively-correlated BSAGs (*BRAP*, *CHCHD5*, *CPT1C*, *GPR1*, *LDLR*, *MAP2K5*, *PLEKHS1*, *SLC30A8*, *ST3GAL2*, *STX16*, *ZFHX3*, *ZGRF1*, *ZNF395* and *ZPLD1*) were associated with “obesity” (Table S4). For instance, SNPs in *BRAP* (BRCA1 associated protein) were shown to associate with obesity and other metabolic abnormalities [41]. *STX16* (syntaxin 16) encoded a protein that is a member of the syntaxin or t-SNARE family, and deletion of this gene caused obesity and macrosomia in humans [42]. Our results revealed that *MAP2K5* (mitogen-activated protein kinase kinase 5) was enriched in the GO cluster “positive regulation of growth (GO:0045927),” and genetic variations in this gene were reported to cause childhood obesity [43]. Furthermore, the evolutionary rates of 14 obesity-related BASGs in large carnivores were higher than those of small ones (Fig. 3). In particular, three obesity-related BSAGs (*ST3GAL2*, *ZGRF1* and *ZPLD1*) were determined to have undergone rapid evolution in extremely large carnivores, although this was not significant in any of the three after FDR correction (Table S6). For instance, the evolutionary rate of *ZPLD1* (zona pellucida-like domain containing 1) is 0.68632 in the extremely large carnivores, 5.5 times that identified in the control group. Deletions in *ZPLD1* were proven to contribute to the genetic susceptibility of common childhood obesity [44]. Mice lacking *ST3GAL2* (ST3 beta-galactoside alpha-2,3-sialytransferase 2) have 50 % more fat mass and 9 % more lean body mass than the control [45]. *ZGRF1* (Zinc finger GRF-type containing 1) encodes a protein that contains GRF zinc finger (zf-GRF) and transmembrane domains, and a recent genome-wide and exome chip association study revealed that the gene is associated with adiposity [46]. Thus, the 14 obesity-related BSAGs identified in this study may contribute to increased body size and accumulated body fat in large carnivores.

It is worth noting that some genes may not show correlation between evolutionary rates and body size across the entire Carnivora phylogeny but instead in some specific lineages within Carnivora. These genes may have been overlooked in the present analysis. Further analysis should be performed in the future to focus on some specific lineages with significant body size variation, which will reveal more mechanisms underlying body size evolution of Carnivora.

### Molecular evidence for Peto’s paradox in carnivores?

Animal gigantism is a recurring phenomenon that seems to be influenced by resource availability and natural selection [19]. Being larger brings organisms an array of advantages, but it also brings biological tradeoffs, including the increased risk of developing cancer due to having more cells [47]. Surprisingly, some studies found that empirical cancer rates do not vary with body size, and large and long-lived animals actually have a lower risk of getting cancer than do smaller, shorter-lived animals; this phenomenon is called Peto’s paradox [20, 22, 48]. In recent years, Peto’s paradox has been studied in many large mammals, such as the bowhead whale and humpback whale [49, 50]. Their genomes provided the following major pieces of evidence related to cancer suppression: (1) multiple duplications of tumor suppressor genes; (2) positive selection in genes related to cancer and aging. It was thus suggested that large species might have evolved multiple mechanisms to suppress cancer.

Carnivores are relatively long-lived mammals, and, generally speaking, species in Pinnipedia have a longer lifespan than do those in Fissipedia. Some extremely large species of carnivores—e.g. walruses and polar bears—were reported to live 40 or more years in the wild [51]. Within certain carnivore taxa, body size and lifespan also seem to be positively-correlated [21]. For instance, sea otters (about 27.4 kg) may live as many as 27 years in the wild, whereas ferrets (about 975.6 g) live a much shorter time—about 11.1 years [21]. The relatively large polar bears (maximum lifespan: 43.8 years) and brown bears (maximum lifespan: 40 years) also live longer than the smaller giant pandas (maximum lifespan: 36.8 years) [51]. However, the molecular mechanism for maintaining longevity in large carnivores is not very clear, and there have been very few relevant studies on cancer in carnivores so far.

In the present study, we identified a total of 100 BSAGs in carnivores that were related to the cancer control process, including tumor suppressor, DNA repair and immunity (Table S5). We do not discuss differences in the expression levels of cancer-related BSAGs among carnivores. The number of cancer-related BSAGs accounted for 29.7 % of the total number of BSAGs, which was far higher than the proportion of cancer-related genes to the total number of functional genes in the human genome (723/21,306, 3.4 %) [52].

Among these cancer-related BSAGs, 21 were determined to be tumor suppressor genes in previous studies (Table 1). For example, *APC* (APC regulator of the WNT signaling pathway) encodes a multidomain protein that plays a crucial role in tumor suppression by antagonizing the WNT signaling pathway. Variants in *APC* would induce various kinds of cancer, such as colorectal and pancreatic cancers [53, 54]. *ZFHX3* (zinc finger homeobox 3) is essential for regulating myogenic and neuronal differentiation, and it was reported to function as a tumor suppressor in several cancers [55]. The evolutionary rates of these two genes are significantly positively-correlated with both body size parameters, suggesting that large carnivores have a higher evolutionary rate than do small ones.

In addition, we identified 15 cancer-related REGs in extremely large carnivores, including two tumor suppressor genes—*ADAM11* and *TEP1*, of which *ADAM11* was still significant after FDR correction. The evolutionary rate of *ADAM11* (ADAM metallopeptidase domain 11) was 0.48527 in the extremely large carnivores, 15.4 times greater than that identified in the background group. This gene was previously identified as a candidate tumor suppressor gene in human breast cancer [56]. Alterations in *TEP1* (Telomerase associated protein 1) were confirmed to cause several types of tumors in humans, including brain, breast, prostate and lung cancers [57]. Both *ADAM11* and *TEP1* had relatively higher evolutionary rates in extremely large carnivores than small ones and suggested an enhanced ability to suppress cancer.

Additionally, 16 BSAGs were found to be related to “DNA repair” (Table S5), and it is well known that deficits in DNA repair capacity might lead to genetic instability and carcinogenesis [58]. A recent study revealed that *HELQ* (Helicase, POLQ-like) plays a critical role in replication-coupled DNA repair, germ cell maintenance and tumor suppression in mammals [27]. Importantly, 18 immunity-related genes were identified in BSAGs of carnivores (Table S5), of which three exhibited elevated evolutionary rates (*MAGT1*, *RFXANK* and *SKAP2*). The evolutionary rate of *MAGT1* (magnesium transporter 1), for example, was 0.33127 in extremely large carnivores, 6.1 times that of the background group. Loss of *MAGT1* disrupts T cell signaling and leads to a novel human primary immunodeficiency [59] and, furthermore, overexpression of *MAGT1* is associated with development and metastasis of colorectal cancer [60]. Here, we obtained 100 cancer-related genes that were significantly associated with body size evolution in carnivores, including 15 cancer-related REGs that were identified in the extremely large group and which might protect the animal from cancer invasion, especially for large and long-lived species. These results might provide novel molecular evidence for Peto’s paradox with regard to carnivores.

### Fixed amino acid changes in extremely small carnivores contribute to growth restriction

There are some extremely small species of carnivores, such as meerkats and ferrets, which have body masses less than 1 kg and body lengths under 50 cm. These two species are distantly related and belong to two suborders (Feliformia and Caniformia, respectively). This small size may allow the species to flourish. For instance, smaller carnivores could take advantage of food resources that are not available to some large animals to ensure survival when the environment changes dramatically and have more free energy and time to engage in activities that increase mating and reproductive success [15–17].

Compared with other carnivores in our dataset, six fixed amino acid changes from six genes were identified in extremely small group (Fig. 4): *CDC7* (S513L), *ENG* (V367I), *LIG4* (S784P), *MMP2* (S579T), *TSPAN8* (S178T) and *POLE* (E701D). These six genes have been shown to be related to the phenotype of reduced body size. While *CDC7* (cell division cycle 7) was highly conserved throughout mammalian evolution, a fixed amino acid change (S513L) was identified in extremely small carnivores. *CDC7* plays essential roles in initiating mitotic DNA replication, and previous study showed that *CDC7*^−/−^ or low expression of the CDC7 protein leads to reduced body size with decreased cell proliferation in mice [61]. Importantly, the fixed changes (S513L) were located in the protein kinase domain that functions as an on/off switch for many cellular processes, including metabolism, cell cycle progression and transcription [62].

Another fixed change (V367I) was examined in the Zona_pellucida domain of *ENG* (endoglin), and it was reported that *Eng* ^−/−^ mice were three times smaller than wild type mice at embryonic day 10.5 of development [63]. The unique amino acid mutation (S784P) was determined in the key domain (DNA ligase IV) of the *LIG4* (DNA ligase 4) gene that was reported mutations in humans or mice would cause growth failure and microcephaly, and this might be the result of activation of the DNA damage response, leading to a large amount of apoptosis during development [64].

A Fixed amino acid mutation was separately found in the *MMP2* (S579T) and *POLE* (E701D) genes. Previous studies have shown that *MMP2* (matrix metallopeptidase 2) knockout in mice and mutations in *POLE* (DNA polymerase epsilon, catalytic subunit) in humans cause short stature [65, 66]. Finally, we found a fixed difference at site 178 of the Tetraspannin domain in *TSPAN8* between extremely small carnivores and others; genetic ablation of this gene in mice caused a reduction (-15.6 %) in the body weight of males fed a normal chow diet [67]. Furthermore, the changes in S513L in *CDC7* and S784P in *LIG4* affect polarity and might cause radical changes in the three-dimensional structure and function of proteins [68]. These six unique changes examined in the extremely small carnivores might have restricted body size growth.

Though body size regulation is an inherently complex process involving many genes and signaling pathways, and not all genes function in the same way in different species and show the same evolutionary pattern, the amino acid sites with fixed changes in different body size groups could still reveal whether the body size variations in different carnivores is driven by common genes or shared mechanism to some degree. Of course, functional experiments are needed in future to further test whether and how these changes cause growth retardation in Carnivora.

## Conclusions

Mammalian order Carnivora exhibits a huge variation in body size—with an over 130,000-fold difference in body mass between the heaviest and lightest species and 50-fold difference in body length between the longest and shortest—but the molecular mechanisms underlying the disparities remain poorly explored. Here we scanned the genomes of 20 representative carnivores and found a total of 337 genes associated with body size. Our analyses showed that 14 obesity-related genes and three rapidly evolving genes might drive body size expansion. The results provided molecular evidence for Peto’s paradox—a lack of correlation between body size and cancer risk—based on 100 body-size-associated genes associated with cancer control and 15 cancer-related genes under rapid evolution in carnivores. By contrast, 15 rapidly evolving genes and unique amino acid changes in six genes might have restricted the growth of small carnivores. This study brings new insights into the molecular mechanisms that drove the diversifying evolution of body size in carnivores, and provides new target genes for exploring the mysteries of body size evolution in mammals.

## Methods

### Phenotype data and orthologous genes

High-quality genomes of 20 carnivores and one outgroup cow (*Bos taurus*) were downloaded from the NCBI database. Eight families in Carnivora (Felidae, Canidae, Mustelidae, Phocidae, Otariidae, Odobenidae, Herpestidae and Ursidae) were chosen to represent a diversity of body sizes. Two kinds of phenotype data—head body length (cm) and body mass (g)—were collected from the PanTHERIA database [69] and used for subsequent correlation analysis. Head body length means the length from the snout of the nose to the root of the tail for an animal [70]. Head body length data were missing on the domestic cat (*Felis catus*) in the database, so we obtained it from the Animal Diversity Web resource [71]. All the phenotype data came from adult individuals.

We then divided these carnivores into four groups—extremely large (body mass > 350 kg), small (body mass < 12 kg), extremely small (body mass < 1 kg) and medium-sized (remaining carnivores)—for subsequent analysis. High-confidence “one-to-one” orthologous gene clusters were identified using the OrthoFinder [72] pipeline, which applied an all-against-all BLSATP algorithm. For genes with various transcripts, the longest coding sequence was used. Transcripts that were shorter than 150 bp or with lengths that were not multiples of three were eliminated by our in-house Perl scripts. The sequences were aligned using Prank [73] at the codon level, and poorly aligned regions with gaps and non-homologous fragments were removed using Gblocks [74] with relatively strict parameters (“-t = c, -b5 = h”). High-quality multiple sequence alignment (MSA) files were used for subsequent analysis.

### PGLS scanning the body-size-associated genes (BSAGs)

PGLS implemented in the “Caper” package in R [75] was used to test the potential association between the evolutionary rates of each gene and each piece of phenotype data (i.e. head body length and body mass). An ultrametric tree of 20 carnivores for PGLS analysis was obtained from the TimeTree website [76]. The Brownian motion model was applied and the phylogenetic signal (λ parameter) was tested by the maximum likelihood (ML) method. The lambda (λ) value was used as a quantitative measure of phylogenetic signals [77]. A λ value estimated to be 1 or near to 1 indicated that these genes showed a strong phylogenetic signal. To obtain more stringent correlation *P* values, we further employed an extra two-step calibration procedure (an alternative to multiple testing correction), as suggested by Ma et al. [78]. Using ‘*P value.all*’ from the regression analysis for all carnivores, two *P* values were calculated: (1) ‘*P value.robust*’ from the regression, repeated after discarding the species with the largest residual error, and (2) ‘*P value.max*’ from the PGLS on the remaining species, to calculate the maximum *P* value after dropping each species on at a time. Genes that were significantly related to both head body length and body mass under the most stringent standard (*P value.all* < 0.05, *P value.robust* < 0.05, *P value.max* < 0.05) are defined as body-size-associated genes (BSAGs).

The evolutionary rate ω—the ratio of non-synonymous (*d*_N_) to synonymous substitutions (*d*_S_)—was estimated using the *free-ratios* model (model = 1) implemented in the CODEML program of PAML 4.9e [79]. The root-to-tip ω of each species was calculated by averaging the ω from the ancestral carnivore to each terminal branch according to the method suggested by Montgomery et al. [39]. If the value of *d*_N_ or *d*_S_ in each ω value is less than 0.0002, then we marked it as an outlier “*n/a*” to prevent it from effecting the integral root-to-tip ω adversely. In addition, all root-to-tip ω values were log_10_-transformed to improve normality for regression analysis [39].

### Functional enrichment analysis

The functional annotation clustering tool Metascape [80] was used to perform Gene Ontology (GO) and KEGG pathway enrichments for the BSAGs list. GO categories were discovered and grouped into annotation clusters against a background of the human genome. All GO terms with an enrichment score (ES) > 1.3 (corresponding to a *P value* less than 0.05) were considered significantly enriched.

We also used literature searches and the GWAS Catalog, Human Phenotype Ontology (HPO; [81]), DisGeNET [82], RefSeq [55], Cancer Gene Census (CGC; [52]), Online Mendelian Inheritance in Man (OMIM; [83]) databases to explore potential biological functions of each BSAG in association with body size.

### Testing for rapidly evolving genes (REGs)

To test whether divergent selective pressures acted on carnivores with contrasting body size based on the BSAGs determined above, we divided the 20 species into three groups: The first was small body-sized carnivores (body mass < 12 kg and body length < 1 m), which included five species: meerkat, *Suricata suricatta*; ferret *Mustela putorius furo*; domestic cat, *Felis catus*; Canada lynx, *Lynx canadensis*; and red fox, *Vulpes vulpes*. The second was extremely large carnivores (body mass > 350 kg), which included four species: polar bear, *Ursus maritimus*; walrus, *Odobenus rosmarus*: northern sea lion, *Eumetopias jubatus*; and Weddell seal, *Leptonychotes weddellii*). The third group comprised the remaining 11 species. A two-ratio model (model = 2) that allows different ω values within the foreground and background branches was used to evaluate the selective pressures in the three groups of species. The small- and extremely large-body-sized groups were regarded as separate foreground branches and the remaining 11 species were regarded as background branches. The null model—i.e. one-ratio model (model = 0)—assumed that all branches have the same ω. The likelihood ratio test (LRT) was used to compare nested likelihood models and the FDR method was used for multiple testing correction (*P adjust*). We defined genes as REGs if their ω of the foreground was higher than that of the background branches with *P value* < 0.05.

### Identification of fixed amino acid changes in small, extremely small and large groups

To explore how changes in single amino acid sites contribute to body size development, we scanned all the orthologous genes set for the fixed amino acid changes in the small group (body mass < 12 kg, i.e. meerkat, ferret, domestic cat, Canada lynx and red fox), the extremely large group (i.e. polar bear, walrus, northern sea lion and Weddell seal) and extremely small species (body mass < 1 kg, i.e. meerkat and ferret) [10]. FasParser [84] was used to pick out the fixed amino acid changes specific to these two groups (compared with other carnivores). For the extremely small group, the stoat *Mustela erminea* (GCA_009829155.1) [85] was added to improve the reliability of the identified amino acid sites. Amino acid sites that were the same in the three extremely small species and were consistently different in other carnivores were selected. Sites containing gaps were also excluded, and positions were corrected using human (*Homo sapiens*) amino acid sequences. Pfam 1.6 [86] was used to determine whether the changes were located in the functional domains of the protein.

## Supplementary Information

**Additional file 1: Table S1.** Genome version and phenotype data for the 20 carnivores used in our study.

**Additional file 2: Table S2.** Significant results for the gene-phenotype association tests for both head body length and body mass (*P value.all/robust/max* < 0.05).

**Additional file 3: Table S3.** GO (Biological Process) and KEGG enrichment results for positively- and negatively-correlated BSAGs.

**Additional file 4: Table S4.** Biological roles of 14 positively-correlated BSAGs that related to obesity.

**Additional file 5: Table S5.** Biological Function of 100 cancer-control-related BSAGs.

**Additional file 6: Table S6.** Rapidly evolving genes in small and extremely large carnivores.

**Additional file 7: Table S7.** All results produced by PGLS analysis and REGs test in this study.

## Data Availability

The data generated and analyzed during this study are included in this article and its additional files, including 8 tables and 4 figures. All genome sequences used in this study are available on the NCBI database (https://www.ncbi.nlm.nih.gov/) under the accession numbers reported in Additional file: Table S1.

## Abbreviations

BLAST: Basic local alignment search tool
BRCA1: Breast cancer type 1 susceptibility protein
BSAGs: Body size associated genes
FDR: False discovery rate
GO: Gene Ontology
GRF: Growth hormone-releasing factor
GWAS: Genome-Wide Association Studies
KEGG: Kyoto Encyclopedia of Genes and Genomes
LRT: Likelihood ratio test
PAML: Phylogenetic analysis by maximum likelihood
PGLS: Phylogenetic generalized least squares
*POLQ*: DNA polymerase theta
REGs: Rapidly evolving genes
SNARE: Soluble NSF attachment protein receptor
SNP: Single Nucleotide Polymorphism
