# Supplementary material for: Genomic insights into body size evolution in Carnivora support Peto’s paradox
Source: BMC Genomics. 2021 Jun 9;22:429. doi: 10.1186/s12864-021-07732-w (PMC8191207; doi:10.1186/s12864-021-07732-w)
Supplement: Supplementary file 4 — Additional file 4: Table S4. Biological roles of 14 positively-correlated BSAGs that related to obesity. [file 12864_2021_7732_MOESM4_ESM.docx]

**Table S4. Biological roles of 14 positively-correlated BSAGs that related to obesity**

| **Gene Symbol** | **Gene Name** | **Roles in Obesity** |
| --- | --- | --- |
| BRAP | BRCA1 associated protein | Polymorphisms in BRAP are associated with overweight, obesity and metabolic traits in human [1] |
| CHCHD5 | coiled-coil-helix-coiled-coil-helix domain containing 5 | SNP rs3748024 in CHCHD5 is associated with body mass index (BMI) and obesity in Chinese children [2] |
| CPT1C | carnitine palmitoyltransferase 1C | CPT1C knockout mice fed a high-fat diet were more susceptible to obesity [3] |
| GPR1 | G protein-coupled receptor 1 | GPR1 is an active chemerin receptor that contributes to the regulation of glucose homeostasis during obesity [4] |
| LDLR | low density lipoprotein receptor | Microsatellite variants in LDLR may play a role in the development of obesity [5] |
| MAP2K5 | mitogen-activated protein kinase kinase 5 | Genetic variations in MAP2K5 were reported to cause childhood obesity and interacted with dietary behaviors [6] |
| PLEKHS1 | pleckstrin homology domain containing S1 | PLEKHS1 is a candidate gene responsible for mild hyperglycemia associated with obesity in rats [7] |
| SLC30A8 | solute carrier family 30 member 8 | Genome-wide association studies have identified SLC30A8 are associated with obesity and type 2 diabetes in Europeans [8] |
| **ST3GAL2** | ST3 beta-galactoside alpha-2,3-sialyltransferase 2 | ST3GAL2-null mice have an increase of 50% in fat mass and 9% in lean body mass [9] |
| STX16 | syntaxin 16 | Deletion in STX16 may cause obesity and Macrosomia in human [10] |
| ZFHX3 | zinc finger homeobox 3 | Polymorphisms in ZFHX3 are associated with obesity in Korean population [11] |
| **ZGRF1** | zinc finger GRF-type containing 1 | A recent genome-wide and exome chip association study was conducted that ZGRF1 was associated with adiposity [12] |
| ZNF395 | zinc finger protein 395 | ZNF395 has a crucial role in the regulation of obesity and the metabolic syndrome induced by hypoxia [13] |
| **ZPLD1** | zona pellucida like domain containing 1 | Deletions in ZPLD1 caused genetic susceptibility of common childhood obesity [14] |

**Notes**: The bold genes exhibit rapid evolution in extremely large carnivores.

**References**

1. Imaizumi T, Ando M, Nakatochi M, Yasuda Y, Honda H, Kuwatsuka Y, Kato S, Kondo T, Iwata M, Nakashima T. Effect of dietary energy and polymorphisms in BRAP and GHRL on obesity and metabolic traits. Obes Res Clin Pract. 2018;12(1):39-48.

2. Wu L, Gao L, Zhao X, Zhang M, Wu J, Mi J. A new risk locus in CHCHD5 for hypertension and obesity in a Chinese child population: a cohort study. BMJ open. 2017;7(9):e016241.

3. Wolfgang MJ, Kurama T, Dai Y, Suwa A, Asaumi M, Matsumoto S-i, Cha SH, Shimokawa T, Lane MD. The brain-specific carnitine palmitoyltransferase-1c regulates energy homeostasis. Proc Natl Acad Sci U S A. 2006;103(19):7282-87.

4. Rourke JL, Muruganandan S, Dranse HJ, McMullen NM, Sinal CJ. Gpr1 is an active chemerin receptor influencing glucose homeostasis in obese mice. J Endocrinol. 2014;222(2):201-15.

5. Rutherford S, Nyholt D, Curtain R, Quinlan S, Gaffney P, Morris B, Griffiths L. Association of a low density lipoprotein receptor microsatellite variant with obesity. Int J Obes. 1997;21(11):1032-37.

6. Lv D, Zhang D-D, Wang H, Zhang Y, Liang L, Fu J-F, Xiong F, Liu G-L, Gong C-X, Luo F-H. Genetic variations in SEC16B, MC4R, MAP2K5 and KCTD15 were associated with childhood obesity and interacted with dietary behaviors in Chinese school-age population. Gene. 2015;560(2):149-55.

7. Kotoh J, Sasaki D, Matsumoto K, Maeda A. Plekhs1 and Prdx3 are candidate genes responsible for mild hyperglycemia associated with obesity in a new animal model of F344-fa-nidd6 rat. J Vet Med Sci. 2016:16-0383.

8. Ng MC, Park KS, Oh B, Tam CH, Cho YM, Shin HD, Lam VK, Ma RC, So WY, Cho YS. Implication of genetic variants near TCF7L2, SLC30A8, HHEX, CDKAL1, CDKN2A/B, IGF2BP2, and FTO in type 2 diabetes and obesity in 6,719 Asians. Diabetes. 2008;57(8):2226-33.

9. Lopez PH, Aja S, Aoki K, Seldin MM, Lei X, Ronnett GV, Wong GW, Schnaar RL. Mice lacking sialyltransferase ST3Gal-II develop late-onset obesity and insulin resistance. Glycobiology. 2017;27(2):129-39.

10. de Lange IM, Verrijn Stuart AA, van der Luijt RB, Ploos van Amstel HK, van Haelst MM. Macrosomia, obesity, and macrocephaly as first clinical presentation of PHP1b caused by STX16 deletion. Am J Med Genet A. 2016;170(9):2431-35.

11. Yang S-A. Association study between ZFHX3 gene polymorphisms and obesity in Korean population. Journal of exercise rehabilitation. 2017;13(4):491.

12. Gao C, Wang N, Guo X, Ziegler JT, Taylor KD, Xiang AH, Hai Y, Kridel SJ, Nadler JL, Kandeel F. A comprehensive analysis of common and rare variants to identify adiposity loci in hispanic Americans: the IRAS Family Study (IRASFS). PLoS One. 2015;10(11):e0134649.

13. Erdenee S, Li J, Kang Z, Xu H, Zang R, Cao X, Yang J, Cai Y, Lan X. Sheep zinc finger proteins 395 (ZNF395): insertion/deletion variations, associations with growth traits, and mRNA expression. Anim Biotechnol. 2020;31(3):237-44.

14. Moleres A, Martinez J, Marti A. Genetics of obesity. Curr Obes Rep. 2013;2(1):23-31.
