# Supplementary material for: Genomic insights into body size evolution in Carnivora support Peto’s paradox
Source: BMC Genomics. 2021 Jun 9;22:429. doi: 10.1186/s12864-021-07732-w (PMC8191207; doi:10.1186/s12864-021-07732-w)
Supplement: Supplementary file 5 — Additional file 5: Table S5. Biological Function of 100 cancer-control-related BSAGs. [file 12864_2021_7732_MOESM5_ESM.docx]

**Table S5. Biological Function of 100 cancer-control-related BSAGs**

| **Function class** | **Gene Symbol** |
| --- | --- |
| Genes associated with Cancer-related phenotypes | ***ADAM11***^a^, *ALPK2*, ***APC***, *ASCC1*^a^, *ASIC1*, ***BRCA2***, ***CDH11***, ***CERS2***, *CLCF1*, *CLSPN*, *DNAJC12*, ***DSC3***, ***DTWD1***, ***EPBH6***, ***ERCC3***, ***ERCC4***, *ETV4*^a^, ***FANCC***, *FLT4*^a^, *FNBP1*, *GDAP2*, ***HELQ***, *HLTF*, ***HRG***, *HSPH1*, *IDH1*, ***ING1***, ***INTS6***, *ITIH3*, *ITK*, *LIG4*, *MANBA*, *MAGT1*^a^, *MMS22L*, *MRAS*, *NR3C2*^a^, *NTRK3*^a^, *PHLDB1*^a^, ***POU6F2***, *PSCA*, *RALGDS*, *RBM15*, *RNF183*, *SEMA3C*, *SERPINE2*, *SLC4A7*^a^, ***STAG1***, ***TEP1***^a^, ***TET1***, *TLN1*, *TNFRSF17*, *TP53I3*, *TRIM66*, *TRIM68*, ***TRMT2A***, *TSHZ2*, *UGDH*^a^, *USO1*, *WDR74*, *WNT2*, *YTDHC2*, *ZBED1*, ***ZFHX3*** |
| Cell adhesion | *ADGRL3*^a^, *CDHR4*, ***DSC3***, *SERPINB8*, *SETD7*, *TENM3*^a^, *UBASH3B* |
| Cell cycle | *APLP2*, *ARAP1*, *BTG4* |
| Cell apoptosis | *AREL1*, *CALHM2*, *MNT*, *RNF183*, *TMEM214* |
| Cell autophagy | *ATG2A*, *EVA1A*, *RETREG3*, *TRIML1* |
| Immunity | *CTLA4*, *DNASE1*, ***EPHB6***, *FER1L5*, *IL12RB2*, *IL17F*, *IL19*, *IL20RB*, *IRF2*, *ITK*, *LIG4*, *LMBR1L*, *MAGT1*^a^, *PPIB*, *RFXANK*^a^, *SKAP2*^a^, *TNFRSF17*, *TNFRSF9* |
| DNA repair | *ASCC1*^a^, ***BRCA2***, *CLSPN*, *EME1*, ***ERCC3***, ***ERCC4***, *FAAP24*, ***FANCC***, *FBXO18*, ***HELQ***, *KMT5B*, *LIG4*, *MMS22L*, *RARP3*, *SSRP1*, *TNKS1BP1* |

Notes: The bold genes represent tumor suppressor genes in cancer and “a” represents cancer-related genes that exhibit rapid evolution in extremely large carnivores.
